# Glutaminase: A Hot Spot For Regulation Of Cancer Cell Metabolism?

**DOI:** 10.18632/oncotarget.208

**Published:** 2010-12-09

**Authors:** Jon W. Erickson, Richard A. Cerione

**Affiliations:** ^1^Department of Chemistry and Chemical Biology, Cornell University, Ithaca, NY 14853, USA; ^2^Department of Molecular Medicine, Cornell University, Ithaca, NY 14853, USA

**Keywords:** cancer, metabolism, glutaminase

## Abstract

Cancer cells re-program their metabolic machinery in order to satisfy their bioenergetic and biosynthetic requirements. A critical aspect of the re-programming of cancer cell metabolism involves changes in the glycolytic pathway (referred to as the “Warburg effect”). As an outcome of these changes, much of the pyruvate generated via the glycolytic pathway is converted to lactic acid, rather than being used to produce acetyl-CoA and ultimately, the citrate which enters the citric acid cycle. In order to compensate for these changes and to help maintain a functioning citric acid cycle, cancer cells often rely on elevated glutamine metabolism. Recently, we have found that this is achieved through a marked elevation of glutaminase activity in cancer cells. Here we further consider these findings and the possible mechanisms by which this important metabolic activity is regulated.

## RHO GTPASES AND CELLULAR METABOLISM IN MALIGNANT TRANSFORMATION

Our initial efforts to identify novel small molecule inhibitors that block malignant transformation were directed at Rho family GTPase-signaling pathways. There were a number of reasons for this, perhaps foremost being that our laboratory has been studying the small GTPase Cdc42, as well as the closely related proteins Rac, and RhoA, and their signaling partners for a number of years. Signals originating from members of this GTPase family have been shown to be important for a broad array of cellular processes ranging from actin cytoskeletal rearrangements to cell polarity, migration, and cell-cycle progression [1]. However, these GTPases have also been implicated in a variety of diseases and developmental disorders, with a number of lines of evidence linking Rho family members to cancer [2]. For example, their hyper-activation as it occurs either through mutations or the de-regulation of their upstream activators, i.e. guanine nucleotide exchange factors which catalyze the exchange of GDP for GTP on these GTPases (such as members of the Dbl family of oncoproteins), results in cellular transformation [3,4]. Cells expressing constitutively active forms of Rho GTPases have been shown to be capable of growing under conditions of serum deprivation and in the absence of a substratum (i.e. anchorage-independent growth), as well as inducing tumor formation when injected into immuno-compromised mice [5-7]. The over-expression of Rho GTPases has been reported in tumors of the colon, lung, and in advanced stage breast cancers, in testicular germ cell and urinary tract tumors, and in pancreatic cancer [8-14]. Two members of the family, RhoA and RhoC, have been implicated in metastasis [15-18], and the expression of the Rho-GTPase-activating protein (Rho-GAP) DLC1 (for Deleted in Liver Cancer 1) is suppressed in liver cancer tissue and in a number of other cancers [19,20]. Thus, collectively these findings make the Rho GTPases and their regulatory proteins attractive candidates for targets of intervention in human cancer.

## A SURPRISING CONNECTION BETWEEN RHO GTPASE-INDUCED CELLULAR TRANSFORMATION AND CELLULAR METABOLIC ACTIVITY

We have recently discovered a new role for Rho GTPases in cancer progression through a previously unappreciated connection to cellular metabolism [21]. In particular, we have found that the hyper-activation of Cdc42 as well as related Rho GTPases (e.g. Rac1, RhoA and RhoC) signals the activation of a mitochondrial enzyme, glutaminase, that plays a key role in glutamine metabolism by hydrolyzing glutamine to glutamate and ammonia. The importance of cellular metabolism in the development of cancer is rooted in the early observations of Warburg that tumor cells exhibit enhanced glycolytic activity (i.e. the “Warburg effect”) [22]. This phenomenon has been receiving a great deal of renewed attention [23-26].

**Figure 1:**
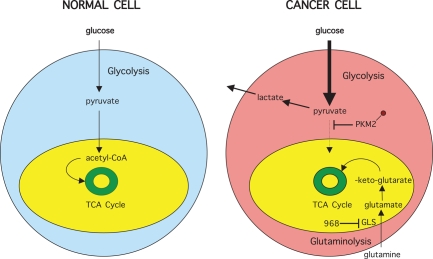
Metabolic remodeling of cancer cells Schematic highlights key differences in many cancer cells compared to normal tissue Normal cells use glycolysis prior to respiration in the mitochondria (yellow) and complete breakdown of glucose by the tricarboxylic acid (TCA) cycle (green). In cancer cells, glycolysis becomes the primary mode of glucose metabolism resulting in lactate and its secretion. The M2 isoform of pyruvate kinase (PKM2) becomes tyrosine phosphorylated and attenuates pyruvate acetyl-CoA conversion while glutaminolysis provides the cancer cell with an alternate source of biosynthetic precursors, fueling the TCA cycle with glutamine-derived a-keto-glutarate. The anti-tumor drug 968 inhibits glutamine metabolism by inhibiting the enzyme glutaminase (GLS).

Cancer cells undergo marked changes in metabolic activity in order to sustain their malignant phenotypes (Figure 1). One such set of changes is the up-regulation of the expression of enzymes in the glycolytic pathway, thus accelerating many of the reactions in this pathway. However, importantly, the penultimate step in glycolysis, the conversion of phosphoenolpyruvate to pyruvate, catalyzed by the enzyme pyruvate kinase, is attenuated (rather than accelerated) in cancer cells [24,25]. This occurs as a result of the tyrosine phosphorylation of a specific isoform of pyruvate kinase (M2) that is preferentially expressed in cancer cells, as well as in embryonic cells, but not in differentiated cells [24,25,27]. The net outcome of this attenuation is that pyruvate is generated through a unique enzymatic mechanism that is uncoupled from ATP production and involves the phosphorylation of phosphoglycerate mutase by phosphoenolpyruvate [26]. Pyruvate, when produced through this “alternative glycolytic pathway”, is converted primarily to lactic acid, rather than acetyl-CoA for citrate synthesis, with citrate then normally entering the citric acid cycle. The increased production of lactic acid by cancer cells, as a result of these changes in the glycolytic pathway, was a seminal observation of Warburg's nearly 80 years ago, and reflects one aspect of the metabolic remodeling that frequently accompanies cellular transformation.

A second major set of changes in cancer metabolism that helps to accommodate the alterations in the glycolytic pathway, results in a shift to increased rates of glutamine metabolism. This occurs through the accelerated hydrolysis of glutamine to glutamate, as catalyzed by mitochondrial glutaminase activity, and the subsequent conversion of glutamate to α-ketoglutarate, catalyzed by glutamate dehydrogenase. The enhanced production of α-ketoglutarate that is the outcome of elevated glutamine metabolism helps to maintain the citric acid cycle in cancer cells, particularly given the loss of the input from pyruvate that is generated via glycolysis in normal (non-cancerous) cells. Metabolic flux experiments using ^13^C-NMR have demonstrated that while proliferating cancer cells exhibit a pronounced Warburg effect, their citric acid cycle remains intact and serves to replenish metabolic intermediates necessary for the production of NADPH for fatty acid synthesis, to provide the carbon source for nucleotide synthesis as well as for the production of asparagine and arginine, and to serve as a major anaplerotic source of oxaloacetate [23,28]. Thus, cancer cells are often referred to as being “glutamine addicted”, as they typically are extremely sensitive to glutamine deprivation and hence cannot proliferate in cell culture without it.

**Figure 2:**
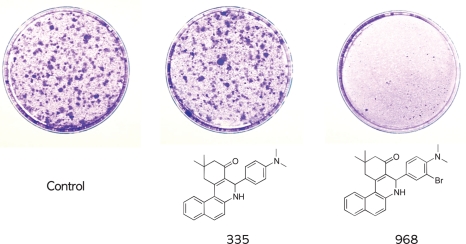
Glutaminase inhibition prevents Rho GTPase driven transformation NIH-3T3 mouse fibroblasts in 10 cm plates were transfected with pZIPneoDbl and allowed to grow for 10 days in the presence of DMSO only (control), 5 M of the inactive 968 analog 335 (CAS registry number 22949-42-4), or 5 M 968 (CAS registry number 311795-38-7), after which the plates were fixed and stained with crystal violet. Structures of the inactive and active compounds are shown below treated cell samples.

Our recent work suggests that the glutamine addiction of cancer cells is enabled by the activation of glutaminase, which catalyzes one of the key steps in glutamine metabolism, as an outcome of post-translational modifications. This discovery stemmed from the screening for small molecule inhibitors that blocked the ability of different constitutively active Rho GTPases, as well as oncogenic Dbl, to transform fibroblasts. These efforts led to our identification of a small molecule inhibitor of Rho GTPase-dependent cellular transformation, designated as 968, that is a member of the benzophenanthridinone family. As shown in Figure 2, 968 was very effective at blocking Dbl-induced focus formation, whereas very subtle changes in the molecule, such as the removal of bromine from the phenyl ring (i.e. molecule 335 in Figure 2), caused it to be ineffective at inhibiting transformation. We went on to show that the target of 968 is glutaminase C (GAC), a specific carboxy-terminal splice variant form of kidney-type glutaminase (GLS1), which is one of two known mammalian glutaminase enzymes (the other being the “liver-type” or GLS2), found in kidney and a variety of other tissues including a number of types of cancer cells [29]. We then demonstrated that targeting GAC, either through the use of the small molecule inhibitor 968 or by RNAi, blocks the growth and invasive activity of various human breast cancer cells, as well as a variety of other types of human cancer cell lines.

We will now consider some of the outstanding questions regarding the regulation of glutaminase activity in cancer cells, and how intervention at the level of this enzyme and glutamine metabolism might offer new avenues for therapeutic intervention against cancer.

## REGULATION OF GLUTAMINASE IN TRANSFORMED CELLS AND CANCER CELLS

There are two obvious mechanisms by which an enzymatic activity might be increased in cancer cells; one is by up-regulating the expression of the enzyme such that its protein levels in cancer cells greatly exceed the levels in non-transformed differentiated cells, and the other is by directly regulating the enzyme's activity. Indeed, it was reported by Gao et al. that glutaminase (GAC) expression was up-regulated in a c-Myc-dependent manner in human B lymphoma and prostate cancer cells [30]. Similarly, we found that the expression levels of GAC were significantly increased in MDA-MB231 cells, a highly aggressive breast cancer cell line, compared to normal mammary epithelial cells [21]. However, at least in the case of MDA-MB231 cells, the up-regulation of GAC cannot fully account for the changes in its enzymatic activity that we observed. Specifically, whereas the basal levels of activity for recombinant preparations of GAC are typically < 5% of the activity measured in the presence of 50-100 mM inorganic phosphate, which serves as an allosteric activator of the enzyme, the basal activity measured in mitochondrial fractions from these breast cancer cells is generally 30-50% of the maximal activity assayed in the presence of inorganic phosphate. Thus, the basal glutaminase activity is disproportionately increased in MDA-MB231 cells compared to the basal activity for the purified recombinant protein. Moreover, glutaminase activity is markedly increased in different transformed fibroblasts and in other cancer cells compared to their non-transformed counterparts, in the absence of any apparent changes in the expression levels of the enzyme. A good example comes from our studies of Dbl-transformed NIH-3T3 cells where the basal glutaminase activity is 5-10 fold higher than the corresponding activity measured in control (non-transformed) fibroblasts, whereas the enzyme activities for these two sets of cells were essentially identical when assayed in the presence of 100 mM inorganic phosphate. Thus, the total levels of glutaminase are essentially equivalent in Dbl-transformed cells compared to control fibroblasts; however, the basal enzyme activity is significantly greater in the transformed cells. The same was found to be true in the human breast cancer SKBR3 cell line.

An even more compelling indication that basal glutaminase activity is increased in transformed/cancer cells, independent of changes in the expression levels of the enzyme, comes from experiments where we ectopically expressed GAC in Dbl-transformed cells and normal fibroblasts. We found that ectopically expressed, epitope-tagged GAC, when immunoprecipitated from transformed cells, exhibited significantly higher basal enzyme activity compared to an equivalent amount of the epitope-tagged enzyme immunoprecipiated from normal cells. These findings provided us with our first clue that glutaminase might be post-translationally modified in transformed/cancer cells and that the modification(s) could explain the activation of its (basal) enzymatic activity, thereby providing a molecular link to the elevated glutamine metabolism exhibited by these cells.

## HOW IS GLUTAMINASE ACTIVATED IN TRANSFORMED/CANCER CELLS?

An important clue regarding how GAC is activated in transformed/cancer cells came from our finding that when the immunoprecipitated enzyme from Dbl-transformed cells was first treated with alkaline phosphatase prior to assaying its enzymatic activity, virtually all of its basal activity (but not the inorganic phosphate-stimulated activity) was eliminated. This suggested that GAC is phosphorylated in transformed/cancer cells and that this is responsible for the marked increase in its basal activity. Transformed cells and cancer cells treated with 968 did not exhibit elevated basal glutaminase activity. Kinetic studies performed using purified recombinant GAC indicated that 968 is neither competing with the binding of substrate (glutamine) nor inorganic phosphate (21). Apparently, 968 acts in an allosteric manner to prevent the activation of GAC in cells by blocking the post-translational modification(s) responsible for activating the enzyme. This would explain how the inhibitory effects of 968 are sustained through the isolation of transformed cells and cancer cells through the washes that are performed to isolate mitochondrial fractions. Moreover, by blocking a post-translational modification(s) that occurs predominantly in transformed/cancer cells, and not in normal (non-cancerous) cells, it is easier to understand why this small molecule inhibitor shows such specificity for transformed/cancer cells in terms of its inhibitory effects.

## THE ROLE OF NFΚB

An important remaining question concerns how the hyper-activation of Rho GTPase-signaling events, which leads to cellular transformation and the induction of malignant phenotypes in various human cancer cells, results in the activation of a mitochondrial metabolic enzyme. Because the increase in basal glutaminase activity occurred in cells transformed by different Rho GTPases (i.e. Cdc42, Rac, and RhoC), and given that each of these GTPases signal through distinct groups of target/effector proteins, we examined whether NFκB might be involved, as it had been reported that a number of Rho GTPases were capable of activating this transcription factor [31,32]. In addition, NFκB was shown to be essential for Dbl-induced transformation and has been implicated in a variety of human cancers, in particular breast cancer [33,34], making it an especially attractive candidate given that a number of different breast cancer cells lines were highly sensitive to 968. In fact, we discovered that blocking NFκB activation with a small molecule that inhibits the degradation of its negative regulator IKBα, as well as knocking-down the expression of the large NFκB subunit, p65/RelA, by RNAi markedly inhibited the activation of glutaminase in Dbl-transformed cells and in breast cancer cells. However, this raises one of a number of pressing questions for the future, namely, how does NFκB activate basal glutaminase activity in transformed/cancer cells?

## QUESTIONS FOR THE FUTURE

NFκB is a well-known transcription factor that has been linked to a wide range of cellular and biological activities including the survival and migration of cancer cells. In the absence of growth factor- or cytokine-signaling activities, NFκB remains in the cytosol associated with a negative regulatory protein IκBα. However, when growth factor/cytokine-signaling pathways are activated, IκBα is phosphorylated and degraded, thereby freeing NFκB to translocate to the nucleus and promote the expression of a number of genes. While the increases in basal glutaminase activity in transformed/cancer cells are dependent on NFκB activity, we know that this is not the result of a direct effect of this transcription factor on glutaminase expression. Indeed, even the enzymatic activity of ectopically expressed GAC is markedly increased in Dbl-transformed cells and this activation is dependent on NFκB. The implication would then be that NFκB is up-regulating either the expression of a growth factor (or cytokine), a protein kinase, and/or a regulatory protein that is essential for the activation of GAC. Recent 2D-gel electrophoresis experiments are consistent with the idea that post-translational modifications of ectopically expressed GAC that are unique to transformed/cancer cells, are responsible for the activation of the enzyme, and that the binding of 968 blocks these activating modifications (J. Wang, unpublished observations). An important goal of our future studies will be to identify the post-translational modification site(s) that leads to the activation of glutaminase in transformed/cancer cells, as this will hopefully provide clues toward understanding the mechanism by which NFκB regulates enzyme activity.

Of course, a number of additional important questions will need to be addressed. In particular, we would like to obtain structural information that sheds some light on how glutaminase is activated in transformed/cancer cells. It has been suggested that GLS1 undergoes an oligomeric transition from an inactive dimer to an active tetramer upon the binding of inorganic phosphate [35]. Does phosphorylation and/or some other type of post-translational modification of GAC help to promote the dimer-to-tetramer transition in cancer cells? If so, is this transition being driven by a combination of the post-translational modification of the enzyme and the binding of inorganic phosphate or another metabolite that might act as an activator in cells? How does the binding of the small molecule 968 block this activation event? Thus far, we have not found 968 to inhibit the ability of high concentrations of inorganic phosphate (i.e. 100 mM) to induce the dimer-to-tetramer transition in GAC. However, 968 might still prevent the enzyme from undergoing this transition in response to lower levels of inorganic phosphate or a specific metabolite, as might otherwise occur in cancer cells not treated with this small molecule inhibitor. These are questions that are now being actively pursued in our laboratory.

## IMPLICATIONS FOR FUTURE THERAPEUTIC STRATEGIES

The fact that inhibiting the activation of glutaminase in transformed/cancer cells with the small molecule 968 has specific effects on the growth, migration and invasive activity of these cells, without affecting the growth or morphology of their non-transformed cellular counterparts, offers exciting new possibilities for therapeutic intervention. The conventional thinking has been that the over-expression of growth factor receptors and their signaling partners gives rise to excessive signaling events that result in malignant transformation and cancer progression [36-38]. Consequently, a number of therapeutic strategies have been directed at inhibiting receptor activation and function, both through the use of tyrosine kinase inhibitors and monoclonal antibodies [39-43]. Some of these strategies have shown some promise, although many of these cancers become resistant to such treatments, and there remains a need for the identification of additional druggable targets that will permit multi-pronged strategies for therapeutic intervention against a number of human malignancies. The ability of the small molecule 968 to alter the unique metabolic fluxes that are necessary for cancer cells to satisfy their biosynthetic and energetic requirements opens the door to novel approaches for intervention against the malignant state. In this regard it is especially noteworthy that 968 blocks the activation of a metabolic enzyme that is selectively activated in cancer. Thus far, the early studies in mouse xenograft models show that the intraperitoneal injection of the small molecule 968 shrinks the tumors in these animals without obvious adverse effects (21). Therefore, targeting glutaminase as well as other enzymes responsible for the metabolic re-programming of cancer cells could offer some exciting and highly specific strategies for intervention against malignancies manifesting glutamine addiction.
